# Heart Rate Variability Dynamics for the Prognosis of Cardiovascular Risk

**DOI:** 10.1371/journal.pone.0017060

**Published:** 2011-02-28

**Authors:** Juan F. Ramirez-Villegas, Eric Lam-Espinosa, David F. Ramirez-Moreno, Paulo C. Calvo-Echeverry, Wilfredo Agredo-Rodriguez

**Affiliations:** 1 Computational Neuroscience, Department of Physics, Universidad Autonoma de Occidente, Cali, Colombia; 2 Engineering Faculty, Department of Automatics and Electronics, Universidad Autonoma de Occidente, Cali, Colombia; Royal Melbourne Institute of Technology, Australia

## Abstract

Statistical, spectral, multi-resolution and non-linear methods were applied to heart rate variability (HRV) series linked with classification schemes for the prognosis of cardiovascular risk. A total of 90 HRV records were analyzed: 45 from healthy subjects and 45 from cardiovascular risk patients. A total of 52 features from all the analysis methods were evaluated using standard two-sample Kolmogorov-Smirnov test (KS-test). The results of the statistical procedure provided input to multi-layer perceptron (MLP) neural networks, radial basis function (RBF) neural networks and support vector machines (SVM) for data classification. These schemes showed high performances with both training and test sets and many combinations of features (with a maximum accuracy of 96.67%). Additionally, there was a strong consideration for breathing frequency as a relevant feature in the HRV analysis.

## Introduction

Cardiac diseases are a major cause of mortality in the world. Studies carried out in 2006 in Colombia establish that heart diseases produced circa 30875 deceases with an overall increase of 19.6% since 1999. Therefore, there has been great interest in the development of computational tools for prognosis and diagnosis. The main aim of these tools is to improve performance of cardiologists on prognostic and diagnostic tasks, i.e., reducing both the number of missed diagnoses or prognoses and the time taken to reach such decisions. Under these conditions, it is expected that detecting cardiac signs helps to decrease the mentioned decease rates. At the same time, the introduction of computational systems offers additional benefits, since the early identification of patients would help the specialists to deal efficiently with certain cardiac diseases. Moreover, as traditional risk stratifiers are commonly used for prognosis, their positive predictive value is not as high as the clinical practice demands.

Heart rate variability (HRV) has been often related with the diagnosis and prognosis of certain cardiac diseases and, in fact, is a standard method for studying the autonomic nervous system (ANS) in heart control [1]. Several findings on HRV analysis have demonstrated that geometric, statistical, spectral, multi-resolution and non-linear approaches are powerful tools for the assessment of cardiovascular health [1], [2], [3], [4], [5], [6], [7], [8], [9], [10], [11], [12], [13], [14], [15], [16], [17], [18]. Several patterns observed from HRV dynamics are often related with myocardial infarction (MI) [18], [19], sick sinus syndrome (SSS) [1], multiple cardiac arrhythmias [1], atrial fibrillation (AF) [20], congestive heart failure (CHF) [21], complete heart block (CHB) [2], and ischemic cardiopaty [2], amongst others. Additionally there are some risk factors that affect HRV directly or indirectly, such as blood pressure (BP), alcohol, smoking and drug consumption [2].

Cardiovascular risk in general terms is often related to low or excessive fluctuations of the NN intervals given by intrinsic and extrinsic factors [10]. The relation between reduced HRV and mortality risk was first shown by Wolf et al. in 1977 [22].

Furthermore, during the last 25 years, the significance of HRV in assessing cardiac health has been recognized and various techniques have been developed in order to analyze the fluctuations of NN intervals. Time and frequency domain analyses are often referred to as classical analysis as they were the very first methods being used for the HRV processing [23], [24], [25], [26], [27], [28], [29]; these methods are often inconvenient as they are linear and stationary methods intending to model a highly non-linear phenomenon. These methods as well as visual assessment of the raw HRV data are the most common approaches used in clinical practice.

In 1996, the Task Force of the European Society of Cardiology and the North American Society of Pacing and Electrophysiology (ESC/NASPE) published standards on HRV analysis. They proposed several time and frequency domain parameters and their clinical uses, based on short-term (5 min) and long-term (24 h) HRV data [12], [21].

Relatively recent findings have shown that frequency domain methods in HRV are related to hypertension [29]. Subjects with risk factors such as hypertension, obesity, insulin resistance, among others, generally show a high sympathetic activity which is often presented before the clinical manifestation of hypertension. As spectral methods are useful to assess the changes in sympathovagal balance, hypertension has been accurately predicted.

Classical methods of analysis are not absolutely suitable for analysis of HRV [21], [30], [31], [32]. Consequently, some approaches have applied multi-resolution methods to HRV analysis. Multi-resolution methods are related to the wavelet transform multi-level decomposition; given the non-stationarity of the HRV signals [31], the discrete wavelet transform (DWT) and wavelet packet transform calculate the required high and low frequency sub-bands, enabling more accurate HRV analysis. Moreover, wavelet entropy measures have been introduced for the implementation of pattern recognition schemes and seem to provide high performances in diagnosing cardiac diseases [21]. However, to our knowledge, previous work has not considered utilizing wavelet energy measures analysis for HRV assessment.

Recently, new dynamic methods of HRV quantification have been used to uncover nonlinear fluctuations in heart rate that otherwise are not apparent. Several methods have been proposed: Return map (Poincaré plot) calculation [13], [14], [15], [33], [34], [35], [36], [37]; Lyapunov exponents/spectrum [2], [38]; 1/f slope [39]; approximate and sample entropy (ApEn and SmEn, respectively) [20], [40]; and detrended fluctuation analysis (DFA) [41]. Moreover, for the last years these analysis techniques have been useful to understand the HRV dynamics as the response of a highly non-linear system, and therefore to produce discriminative enough features to reach high success rates when several pattern recognition techniques are implemented [2], [20]. Pattern recognition in HRV has been used for a variety of applications from prognosis to diagnosis of heart diseases. The most commonly used schemes include: Artificial neural networks (ANN) frames [20]; support vector machines (SVMs) [1], [20]; and linear statistical classifiers [21]. In general terms, the performance of these classifiers in prognostic or diagnostic tasks is relatively high (80% to 95% sensitivity in the best cases); however, they have been used for the recognition of several patterns in specific cardiac diseases (e.g., CHF, paroxysmal AF, MI, cardiac arrhythmias, amongst others) rather than for the prognosis of cardiovascular risk.

In this work, HRV analysis methods and pattern recognition schemes (namely, artificial neural networks and support vector machines) were used to discriminate between healthy control subjects and cardiovascular risk patients. Extensive experiments were carried out regarding the overall usefulness of the features with emphasis on the prognostic values associated to classical and non-linear analysis methods. We determined the potential application of such methods to clinical practice in order to increase the success rates of cardiovascular risk assessment. There is a strong consideration for breathing frequency as a relevant feature of the HRV analysis, given the respiratory sinus arrhythmia (RSA) phenomenon [25], [42], [43], [44]. Additionally, we provide a brief explanation on the implementation of advanced HRV analysis software using the analyses performed in this work and for automatic cardiovascular risk prognosis.

## Materials and Methods

### 1. Ethics Statement

This study was approved by the Institutional Review Board of Universidad Autonoma de Occidente (UAO), Cali, Colombia. Each patient in this study was informed in detail about the procedure and signed an informed consent which guaranteed the transparence of the test and the records' future usage.

### 2. ECG Database

Two distinct source materials were employed in this study: (a) A database of risk and non-risk individuals given by Coomeva IPS (Health Provider Company) experts and (b) the corresponding electrocardiographic data extracted by us using a medical expert protocol.

The requirement for patient's clinical history and positive or negative cardiovascular risk verification was assessed by Coomeva experts as this verification concedes extreme importance on the validity and quality of the subsequent results of this work. The main purpose of this step was to measure the relationship between HRV indices and the subjects with risk factors. Diabetes Mellitus, high blood cholesterol and other lipids, high blood pressure, metabolic syndrome, overweight, obesity, physical inactivity, tobacco and drug consumption are common risk factors of cardiovascular heart diseases (CHD) and heart failure; nearly all of these risk factors are associated with HRV reduction or excessive fluctuations [45], [46], [47], [48], [49]. All the risk subjects (patients) showed at least 3 significant risk factors according to the experts' risk assessment.

In order to get the ECG and respiratory signals, a PowerLab device -ref. ML865- (ADInstruments) and a piezoelectric band -ref. MLT1132/D- were used. PowerLab is a data acquisition system used in a variety of experiments and applications with humans' biopotentials. The unit can record more than 200000 samples per second and has individually selectable input sensibilities. Additionally, it has a bioamplifier (used to record any biological signal from the human body or other source) and an internal processor with low and high pass filters. The hardware of the PowerLab uses the software package Chart and Scope in order to record and analyze each acquired dataset.

On the other hand, a non-invasive blood pressure (NIBP) measure was taken into account for the HRV evaluation as it is considered one of the main risk factors in the cardiovascular assessment [49]. Such measure was extracted by using a multiparameter monitor Spacelabs ref. 90309 which allows us to monitor the following parameters: Electrocardiography, respiration, temperature, non-invasive blood pressure and pulse oximetry.

Each register (a total of 90 electrocardiographic records) was recorded following a medical protocol designed by the teamwork using the frontal-bipolar derivation (D2) of the electrocardiogram. The duration of each record was 5 minutes, following the international standards established by the Task Force of the ESC/NASPE [12]. In addition, the following information was taken from each patient: Age, gender, weight, height, diagnosed cardiovascular diseases, risk valuation given previously (without risk, medium risk or high risk), diagnosed risk factors. All the subjects of this study were in sinus rhythm during the ECG recordings, furthermore, the mean breathing frequency for all was 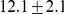
breaths/min.

#### 2.1. HRV and Respiratory Sinus Arrhythmia Considerations

Respiration has an important influence in HRV. This phenomenon is known as respiratory sinus arrhythmia (RSA), which is a rhythmic fluctuation of the heart beat intervals in a phase relation with the inspiration and the expiration. The autonomous nervous system (ANS) is the part of the nervous system which extrinsically controls the vital functions and organs such as the heart, lungs and glands. ANS is divided into two major subsystems: The Sympathetic Nervous System and the Parasympathetic Nervous System. These systems are antagonists and responsible for the tuning of some physiologic mechanisms. Intrinsic as well as extrinsic factors may affect such balance and sometimes provoke different nervous activity patterns (sympathetic or parasympathetic), which are often the cause of functional irregularities; those patterns are given by hyper/hypoactivity of such subsystems.

When the respiration is being monitored in a controlled environment, the R-R intervals tend to be shorter during the inspiration and larger during the expiration. Various theories have been exposed about this phenomenon, according to many experiments on animals [43]. The RSA comes from the control given by the oscillations in the firing rates of the medullar neural networks (central pattern generators, CPGs); the medullar neural network shows periodic oscillations even when the afferent inputs are interrupted. When those oscillations are carried out by afferent stimuli from the receptors in the lungs and the thoracic wall, there is a cardiac rhythm oscillation known as RSA.

Obtaining the respiratory frequency has been used to evaluate the magnitude of the RSA, defined as the sum of the power spectral density estimations on the respiratory band. The variations induced by the respiratory rate introduce a significant change on the measurement of HRV; however, if the respiratory frequency is a constant (i.e., 12 breaths/min) and the tidal volume is relatively constant for the maximum capacity, the error in the measurement caused by respiratory irregularities is eliminated and allows a better stability and an effective comparison of the RSA magnitude between patients [50]. In this study, the above criteria were used to measure HRV in the patients. Consequently, both the recorded ECG signals in resting conditions and specific conditions of the environment were taken into account for the vital signs stabilization.

### 3. ECG Data Pre-processing

#### 3.1. R-Peak Extraction

The R peaks were extracted using the Pan-Tompkins algorithm and the wavelet transform by keeping the detail coefficients from 2^1^ to 2^4^ using the Haar wavelet [51].

In order to know the effectiveness of the wavelet decomposition levels, the performance of the algorithm was measured using the detail coefficients from the first decomposition level to the fourth decomposition level using on 6120 heart beats recorded with the implemented medical protocol. The results of this evaluation procedure are summarized in Table 1.

**Table 1:** Performance of the R-peak detection algorithm at various wavelet decomposition levels (sampling frequency of 200 kHz) using the Haar wavelet.

Decomposition Level	FP	FN	Sensitivity (Se - %)	Accuracy (Ac - %)
1	240	270	96.06	91.98
2	220	0	96.53	96.53
3	50	0	99.19	99.19
4	0	0	100.00	100.00

According to Table 1, the most appropriate wavelet decomposition level to extract the R-peak is 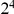, with a sampling frequency of 200 kHz for the ECG signal and using the Haar wavelet. However, the diverse resolution levels on the R-peak extraction procedure may affect the temporal resolution of the signal; thereby a comparison of the estimation of the R-R series was made using the resolutions taken into consideration for this work. We found that, despite the detected false positives, there are no significant spatial and frequency changes on the signal given by the decimation process; thereby, there are no significant variations on the spectral indexes estimation.

#### 3.2. Outlier Removal Procedure

Past publications have shown that eliminating the ectopic HRV data is often better than interpolating them or doing any other cumbersome procedure [52]. Grubbs Test extended by Rosner method was used in this work [53], [54]. Assuming a normal distribution, Grubbs' outlier test can be used to remove one outlier. Nevertheless, if we decide to remove this outlier, we might be tempted to run Grubbs' test again to see if there is a second outlier in the data; however, the rejection criteria changes. Rosner has extended Grubbs' method to detect several outliers in one dataset. Rosner's several outliers detection method seems to be compatible with HRV signals in general ways [54].

For a specified limit 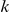 of the number of outliers, the procedure is calculated by using reduced samples of length 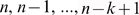, respectively. For each sample 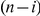:
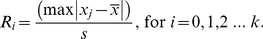
(1)


where 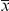 is the mean and 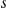 is the standard deviation of the sample 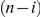 and *j* is the position of one given value of the sample. In this way, the critical values of the test are determined by specifying 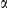 and by calculating 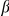 and 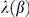 in order to calculate the *t*-student statistical test [53].

### 4. HRV Classical Measures

#### 4.1. Statistical or Time-domain Measures

In this approach, a set of 7 well-known statistical indexes were calculated: First, the mean and the standard deviation (SDNN) of the NN intervals of each 5 min record; second, the square root of the mean of the sum of the square of differences between adjacent NN intervals (RMSSD); third, the so-called pNN50 was computed as the NN50 count value divided by the total of all NN intervals, where NN50 is the count of adjacent intervals differing by more than 50 ms in the entire HRV record; fourth, the interquartile margin of the NN intervals (MIRR) was also calculated, i.e., the first quartile subtracted from the third quartile of the NN series; in addition, the median of the absolute differences of the NN intervals (MDARR) and the standard deviation of the differences between adjacent NN intervals (SDSD) were calculated.

#### 4.2. Spectral or Frequency-domain Measures

Spectral or frequency-domain measures are based on the power spectral density (PSD) analysis of the R-R series. In this kind of analysis some processing techniques, such as interpolation and detrending, are necessary. The spectral measures have the advantage of relating the power of variation in different frequency bands to different physiological modulating effects [2], [24]. Extensive experiments have shown that parametric methods (AR spectrum) tend to produce better results than classical nonparametric methods (Welch's periodogram) when the data length of the signal is relatively short, as is the case with HRV data [20]. For this reason, we applied parametric PSD estimation. Three main spectral measures are distinguished from the spectrum of the R-R series: The power of the very low frequency band (VLF band, 0–0.04 Hz), the power of the low frequency band (LF band, 0.04–0.15 Hz) and the power of the high frequency band (HF band, 0.15–0.4 Hz). These frequency components and their normalized values (NLF and NHF) were calculated using a standard integration procedure (area under the curve) of the spectrum regions. In addition, the ratio of LF to HF was calculated as it indicates the balance of ANS.

For parametric spectral methods, the data can be modeled as the output of a discrete and causal filter whose input is white noise.

The spectral power of an AR process is given by:
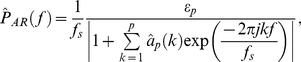
(2)


where 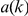 are the recursive coefficients calculated by covariance method [2]. An important factor in the implementation of the AR method is the selection of the order [28]. The order *p = 16* for the AR model was taken into account for this study [26], [54], [55].

### 5. Wavelet Packet Measures

According to the literature consulted [21], there is a three step procedure to calculate the wavelet measures: First, calculate the wavelet packet coefficients; second, calculate the wavelet energy; and third, calculate the wavelet entropy.

The wavelet packet analysis in HRV is used to separate the signal into multiple scales. This method allows us to analyze both frequency and spatial domains and removes polynomial non-stationarities of the signal [11]. Due to this property, wavelet analysis is much more suitable for analyzing HRV signals than statistical and spectral methods. In this work, the wavelet packet analysis was implemented by using the DB4 function as mother wavelet [21]. The decomposition was performed at a level of 5 [21], [56], [57].

Once the wavelet coefficients are known, it is possible to calculate the energy for each coefficient: 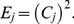
(3)


Then, the total energy can be calculated as the mean value of the energy for each coefficient.

On the other hand, the wavelet entropy can be calculated as the probability distribution of the coefficients or the wavelet energy into normalized values:
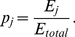
(4)


In this way, using the definition of entropy given by Shannon, the entropy can be calculated as follows:
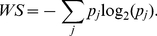
(5)


In [21], the wavelet entropy is calculated as a unique multi-resolution measure. However, in this work, both the energy and entropy measures are taken into account due to their high cardiovascular risk prognostic value.

A problem with wavelet packets implementation is coping with redundant information coming from the wavelet transform computation for the approximation and detail coefficients [54]. In order to avoid the redundant information due to wavelet packet decomposition, a standard Principal Component Analysis (PCA) was computed. PCA chooses a dimensionality reduction by linear projection that maximizes the scattering of all projected samples. The resultant feature space is the projection of the original data set over the covariance matrix eigenvectors, which constitutes the weights of each feature on the input space according to how much of the model's variability is explained by them. The most important entropy and energy features (in terms of variability) were selected.

### 6. Non-Linear Measures

HRV has been often evaluated using non-linear methods [6], [20], [58]. These methods seem to be very relevant in feature extraction of the HRV series; on the other hand, HRV dynamics is highly non-linear and, actually, HRV series is the response of a chaotic system, i.e., a system with high sensitivity on the initial conditions [59], [60], [61].

#### 6.1. Poincaré Map-Based Features

The most popular non-linear technique to assess the HRV is the so-called Poincaré map (also called return map or Lorenz map). The Poincaré map corresponds to the reconstruction of the attractor of the system based on the HRV experimental series [60]. This map can be constructed by plotting each RR interval against the next interval. This plot is very useful in summarizing beat-to-beat information on heart behavior. The Poncaré plot is a simple visual interpretation technique and it has proved to be a very powerful predictor of disease and cardiac dysfunction [21].

In order to extract features of the Poincaré map, two methods were applied in this work: The ellipse fitting technique and the histogram technique [17].

A group of axes with orientation onto the identity line or principal diagonal is the main feature of the ellipse fitting technique. The axes of the plot are related to a new group of axes with a rotation of 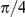 [17].

On the new reference system axes, the dispersion of the points through the 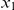 axis is measured by its standard deviation, denoted SD1. On the other hand, the magnitude of the points through the identity line shows the level of long term variability, denoted SD2, i.e., the standard deviation over the 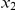 axis.

On the other hand, the ellipse approximation is satisfactory in many cases; however, the shortening that occurs on short R-R intervals is not taken into account by this technique [35]. The histogram approximation has been used to evaluate the distribution of the data into several time ranges. There are three types of histograms: The width histogram, the NN interval histogram and the length histogram [17].

As the visual interpretation of the histogram can be useful to extract information about the heart, it is necessary to parameterize it. The computation of the width of the three histograms is a very strong feature for the prognosis of cardiovascular risk, as it gives the absolute statistical ranges of the NN intervals and its projections. These features are useful for assessing short term HRV, long term HRV and the distribution of NN intervals itself. These histogram widths were taken into account in this study.

#### 6.2. Complexity Analysis

There are several approximations for estimating regularities of different kinds of signals. The most widely used complexity measures for short and noisy data series are approximate entropy (ApEn) and sample entropy (SmEn). These features assign a non-negative number to temporal series in order to quantify the regularity of its fluctuations. Given this fact, complexity measures have been highly useful for the analysis of HRV signals [20].

To calculate ApEn and SmEn from temporal series it is necessary to choose two parameters: A length *m* and a window size *r*. ApEn measures the logarithmic similarity amongst neighboring input patterns (those with a separation radius less than *r*) for *m* contiguous observations. On the other hand, SmEn is an unbiased estimator introduced to avoid the self-couplings and to quantify the regularity of highly irregular temporal series. SmEn is equal to the negative of the natural logarithm of a conditional probability. It is the probability that sequences close to each other for *m* consecutive data points will also be close to each other when one more point is added to each sequence [20].

For both, ApEn and SmEn calculation, it is recommended to take 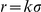, where 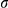 is the standard deviation of the data series and *k* runs over 0.1 to 0.2 [62] (for details regarding the calculation of complexity analysis measures see Appendix S1).

### 7. Statistical Significance Tests and Feature Selection

**Figure 1:**
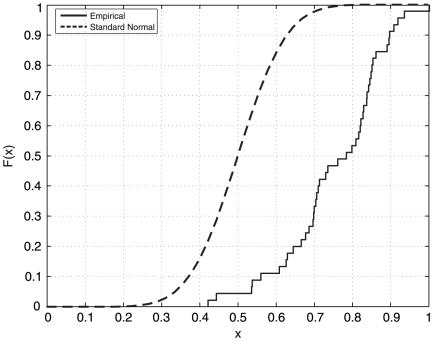
Cumulative distribution plots comparison between a given feature considered in this work and the standard normal distribution.

Up to this moment, several statistical methods have been related to feature selection for training and testing classifiers and for improving their overall performance. As most of computational methods in pattern recognition require a feature selection step, the nature of such procedure depends largely on the structure of the data. As a result, many computational approaches use parametric statistics, e.g., the so-called multivariate analysis of variance (MANOVA), which often reports adequate results for such purposes. However, we cannot assume that all the data has resemblance to a standard normal distribution at high statistical significance. Therefore, we implemented two methods in order to test the normality of the data: First, we conducted the Pearson's chi-square test; and second, the one-sample KS-test. According to our results, the data (for all the features taken into account), has a remarkably different distribution when compared to the normal distribution (with 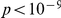). As both methods reported quite similar results, we concluded that non-parametric (distribution-free) statistical methods needed to be implemented at this stage. These methods, unlike parametric statistics, make no assumptions about the probability distributions of the variables being assessed. Figure 1 depicts the empirical cumulative distribution plot for a given feature (note the significant difference to the standard normal distribution).

Statistical significance of these results was tested using a standard two-sample KS-test. A level 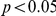 was considered a statistically significant difference [63]. On the other hand, in order to use the best features on the classification stage, a level 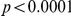 was considered statistically relevant enough as KS test-based selection criteria.

### 8. Classification

Multilayer Perceptrons (MLP), Radial Basis Function (RBF) networks and several Support Vector Machines (SVM) were evaluated for the classification stage of this work. All classification schemes were trained to capture the difference between cardiovascular risk subjects and healthy ones.

#### 8.1. Multi-layer Perceptron (MLP) Neural Network

Multilayer Perceptrons (MLP) are frequently implemented for classification tasks, given their generalization capabilities. In this work, a standard three-layer network has been proposed.

Let *μ *(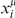) be an input pattern, the output of a single artificial neuron of the hidden layer is given by the following equation:
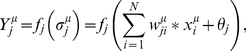
(6)


where 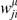 is the synaptic weight *i* of the neuron, 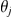 is the bias and 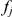 is the activation function. In the current model we have two non-linear transfer functions corresponding to the hidden and the output layers, given by the following equations, respectively.
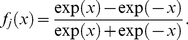
(7)

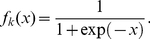
(8)


This architecture guarantees that the network's output will run over 0 and 1 (given the sigmoid output function).

We used the Levenberg-Marquardt backpropagation algorithm to train the MLP neural network, as it is the most popular and successful learning method for training MLPs. The algorithm employs iterative mean squared error minimization using least squares curve fitting [64].

The network consisted of three layers (the sensory input layer, the hidden layer and the output layer), with 5, 200 and 1 neurons, respectively.

#### 8.2. Radial Basis Function (RBF) Neural Network

RBF network is a well-known classifier which combines supervised and unsupervised learning. A standard three-layer RBF network was implemented in this work. The hidden layer of the network is responsible for producing a non-linear expansion of the input space to a hidden space where the classes are linearly separable by unsupervised learning [64]. The most popular unsupervised learning rule for the hidden layer is the so-called *k*-means.

In this procedure, we establish a number of neurons (*k*), whose synaptic weights 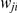 are randomly distributed on the input space and then a similarity measure is calculated, i.e., the Euclidean distance.

When this procedure is applied to the whole layer, the weight update rule is then calculated from:
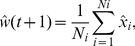
(9)


where 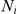 is the number of input vectors associated to each Gaussian node of the hidden layer. This procedure is computed until the stabilization of the synapses is reached (the weights do not change from a training cycle to another).

The Gaussian scale parameters for each hidden neuron can be determined by the approximated magnitude of the influence radius of each neuron on the input space in relation to other neurons near to the *j* neuron.

This network consisted of three layers (the sensory input layer, the hidden layer and the output layer), with 5, 3 and 1 neurons, respectively.

#### 8.3. Support Vector Machines (SVMs)

In the most cumbersome case, the patters are not linearly separable. The main objective of the SVM schemes is to map the input data from the N-dimensional space to the M-dimensional space (M>N), where the classes are supposed to be linearly separable and can be classified by the calculation of a standard separating hyperplane [64], [65] (see Appendix S2 for details on the implementation of SVM).

In our work, polynomial and radial basis function (RBF) as well as linear SVM were used to classify the input data.

#### 8.4. Normalization, Validation and Performance Measures

In order to train the classifiers and perform the KS-tests, all samples were normalized using the MinMax normalization [21].

There are several ways to compute the performance of a recognition system. The pattern recognition schemes were evaluated using two different procedures: The calculation of several performance measures such as sensitivity (Se), specificity (Sp), positive predictive value (Pp), negative predictive value (Np) and accuracy (Ac), all in the interval [0.00,100.00]. On the other hand, Receiver Operating Characteristic (ROC) curve was used in order to measure the accuracy of ANNs. The ROC curve is the plot of the true positive rate (Se) versus the false positive rate (1 – Sp) for different testing points in a diagnostic test. An ROC curve illustrates various aspects: First, it shows the tradeoff between the sensitivity and the specificity in the evaluation of a model; and second, it is a measure of the accuracy of the algorithm given by the area under the curve, i.e., the algorithm's probability of giving correct classifications when a new input pattern is presented [29].

### 9. Computational Implementation

**Figure 2:**
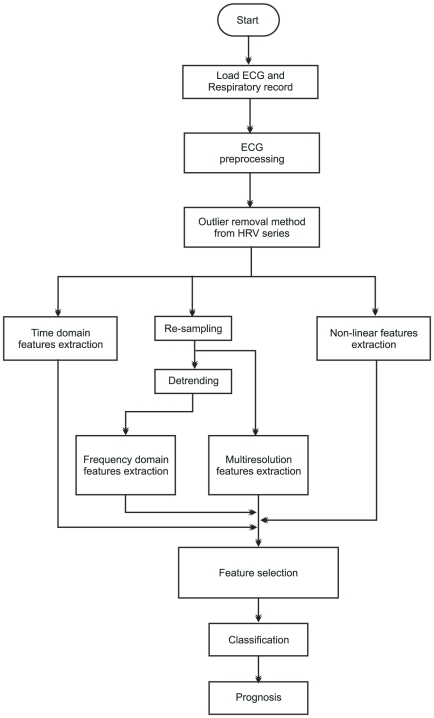
Flow diagram of the computational implementation of the computational tool reported in this work.

For each subject statistical, spectral, multi-resolution and non-linear features were calculated using Matlab™ 7.6.0. The flow diagram of the whole system is depicted in Figure 2.

The application works with 5-min electrocardiographic (ECG) signals; thereby, a preprocessing step is involved in the procedure, i.e., the R-peak extraction and NN intervals calculation. Therefore, there is an ectopic beat removing algorithm, i.e., Grubbs Test extended by Rosner outlier detector. The next stage contains the feature extraction, i.e., statistical, spectral, multi-resolution and non-linear features calculation. For spectral features calculation, the 4 Hz cubic interpolation and the smoothness priors 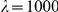 as detrending method are performed in the whole HRV dataset. The wavelet packet-based features are extracted using DB4 mother wavelet and the decomposition is done to a level 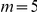 [21]. Ellipse fitting and histogram features are extracted from the first-order Poincaré plot. SmEn and ApEn complexity measures are extracted using 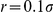 and 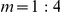. All the features mentioned above can be displayed by the user. Feature selection is performed via KS-tests and the top-5 features are used to distinguish normal subjects (N) from cardiovascular risk ones (R). The classification scheme is responsible for giving the final prognosis result.

The respiratory rate mean and standard deviation feature can be used to corroborate that respiratory rate is relatively constant over the whole ECG record and that is approximately equal to 12 breaths/min. If this condition is not fulfilled, the results would be invalid.

## Results

### 1. Statistical, Spectral, Multi-resolution and Non-linear Analysis of Extracted HRV Data

All the feature analysis results obtained in this work were reported using standard box diagrams, given their suitability for this statistical analysis and since their interpretation can be performed in a remarkably easy way, regardless of the fact that variables could present a great deviation from the normal distribution. The tops and bottoms of each box are the 25^th^ and 75^th^ percentiles of the samples, respectively. The line in the middle of each box is the sample median; this illustrates the skewness of the samples. The dashed lines extending below and above each box are drawn from the ends of the interquartile ranges to the furthest observation within the dashed line length. Crosses are the outliers of the samples; they represent atypical data sufficiently distant from the limits of the box. Note that their elimination is not justified, provided that the objective of box diagrams is to give a complete knowledge of the shape of the data distribution.

**Figure 3:**
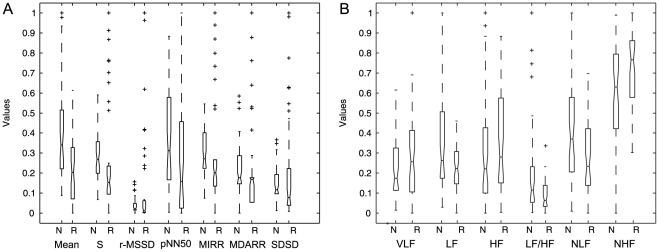
Box diagrams. (a) Statistical measures and, (b) Spectral measures from 5-min HRV records from normal (N) and risk (R) subjects in normalized values (*y* axis).

Statistical, spectral, multi-resolution and non-linear features were calculated from the recorded HRV database. The statistical analysis for the classical measures is shown in Figure 3.

The KS-test showed that all statistical features (mean, standard deviation, RMSSD, pNN50, MIRR, MDARR and SDSD) are statistically significant (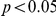) for the comparison of normal (N) and cardiovascular risk (R) subjects. The most statistically significant features were the standard deviation, RMSSD, MIRR and SDSD (with 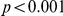); the remaining features reported significances close to the alpha value.

In addition, LF power, LF/HF ratio, NLF power and NHF power showed statistically significant differences (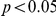) between normal and cardiovascular risk subjects; however, VLF and HF powers do not discriminate between these two groups (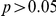).

**Figure 4:**
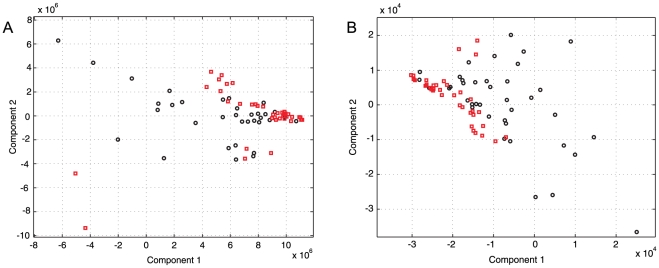
PCA transform of multi-resolution features of the 5-min HRV records from Normal (black diamond) and cardiovascular risk (red square) subjects. (a) entropy features and, (b) energy features.

Standard PCA was applied to the multi-resolution measures in order to obtain the most relevant features in terms of variance. The total of selected groups of wavelet coefficients was 26 out of 62 (the total of wavelet packet coefficients from a decomposition level of 5). These 26 groups of coefficients, according to the PCA, retain approximately 98% of the variance of the model; however, the features projections given by this transformation were not used to train the classifiers due to the decreasing statistical significance of the projected features. The main results of PCA for the two principal components are illustrated in Figure 4. The second principal component projection showed statistical significance in both energy and entropy wavelet features; these two features retain approximately 50% of the variance of the model. In this analysis, even the third component showed statistical significance; however, the rest of the projected data were not significantly different or discriminative between normal and cardiovascular risk subjects.

A total of 27 wavelet packet-based features were selected. Statistical significance levels of the entropy measures showed that 15 wavelet entropy components were statistically significant with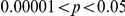 (1 from the first decomposition level, 2 from the second one, 1 from the third one, 3 form the fourth one and 8 from the last one). On the other hand, the significance levels for energy features showed that 12 wavelet energy components were statistically significant with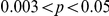 (1 from the first decomposition level, 1 from the second one, 2 from the third one, 3 form the fourth one and 5 from the last one). The remaining entropy and energy components were not taken into account because they did not discriminate between the two groups (normal and cardiovascular risk subjects) with statistical significance.

**Figure 5:**
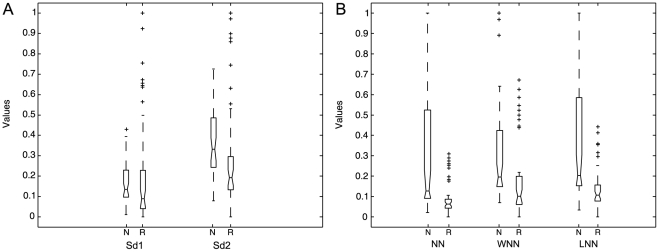
Box diagrams of Poincaré map-based features. (a) SD1 and SD2 ellipse fitting features and, (b) NN histogram (NN), width histogram (WNN) and length histogram (LNN) features of the 5-min HRV records from normal (N) and risk (R) subjects in normalized values (*y* axis).

Non-linear analysis KS-test results are illustrated in Figure 5. The results of the non-linear analysis showed that SD1 and SD2 ellipse fitting features are statistically significant (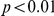, in the best case); SD1 being the less significant one. Additionally, there is statistical difference in histogram technique parameters, i.e., the widths of the NN intervals, the width and the length histograms of the HRV records, among normal and cardiovascular risk subjects. According to the statistical analysis, statistical significance increases with the NN intervals histogram width (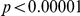) and the length histogram width (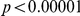). For the case of the width of the width histogram the statistical difference is relatively high (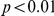).

**Figure 6:**
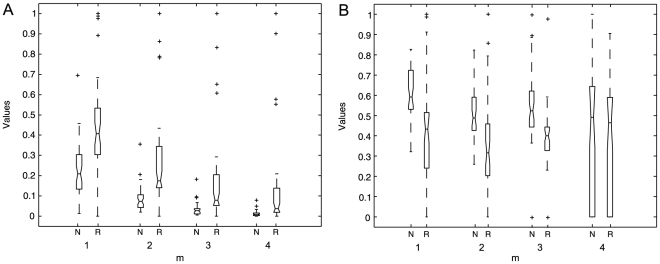
Box diagrams of complexity measures. (a) ApEn and, (b) SmEn (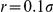 and 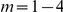) of the 5-min HRV records from normal (N) and risk (R) subjects in normalized values (*y* axis).

ApEn and SmEn significance test results are contained in Figure 6. ApEn shows increased statistical significance (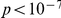) for 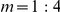. On the other hand, SmEn shows statistical significance (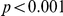) only for 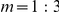, for 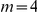 there is no statistical significance for the comparison of normal (N) and cardiovascular risk (R) subjects. Table 2 contains the 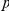 values of ApEn and SmEn at various 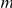 values; the 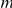 value varies from 1 to 4. The subsequent values did not allow obtaining statistically significant ApEn and SmEn values due to the relatively short duration of the HRV records (5-min per HRV record) [20].

**Table 2:** Statistical significance for complexity measures at different *m* values.

m	p-value (ApEn)	p-value (SmEn)
1	1.87E–08	2.38E–07
2	1.21E–09	1.60E–04
3	4.87E–09	2.52E–06
4	6.83E–08	0.6101

The total number of features extracted from spatial and frequency domains, multi-resolution and non-linear algorithms is equal to 52 (7 statistical, 6 spectral, 27 multi-resolution and 12 non-linear features). The total number of optimal features extracted by KS-tests is equal to 5 due to the statistically significant discrimination presented by them (the next section will illustrate this fact clearly). The resulting feature set combined 3 non-linear and 2 multi-resolution features. These features were used to train and test the ANN and SVM classifiers. Additional experiments were conducted in order to compare the performance of the four principal PCA feature projections to those chosen by KS-tests.

### 2. Artificial Intelligence Schemes Classification for the Prognosis of Cardiovascular Risk

#### 2.1. KS test-based Feature Selection Results

In order to evaluate each artificial intelligence (AI) scheme, the cross validation method was used. This method allows generating the indexes for the validation of the *N* observations by choosing randomly the training and test observations. Each AI scheme was trained using approximately 66% of the observations (60 HRV records, 30 from normal subjects and 30 from cardiovascular risk subjects) and tested using the remaining 33% of them (30 HRV records, 15 from normal subjects and 15 from cardiovascular risk patients). This division gave an optimal performance (high generalization levels) for the ANNs as well as for SVMs.

**Figure 7:**
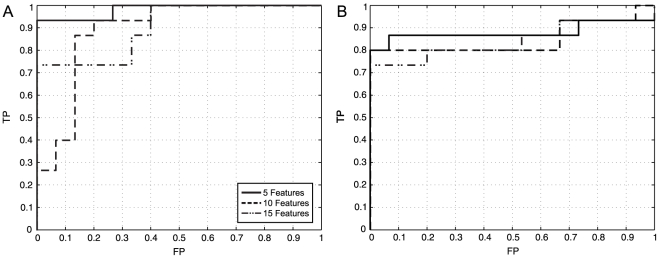
ROC curves. (a) For MLP neural networks, (b) RBF neural networks obtained from the classification of the HRV test records using 5, 10 and 15 top features selected by KS-test; the areas under the curves are equal to 0.9822, 08889 and 0.9024 for MLP and 0.8800, 0.8667 and 0.8400 for RBFNN, respectively.

Experiments were performed using 5 (3 from non-linear domain and 2 from multi-resolution domain), 10 (4 from non-linear domain, 3 from multi-resolution domain and 3 from statistical domain) and 15 features (5 from non-linear domain, 6 from multi-resolution domain and 4 from statistical domain) selected by KS-test. The results of MLP and RBF neural networks as well as of SVM classifications of HRV records from normal (N) and cardiovascular risk (R) subjects are illustrated in Table 3. The ROC curves for both neural network schemes are depicted in Figure 7; the areas under the curve are 0.9822, 0.8889 and 0.9024 for MLP; and 0.8800, 0.8667 and 0.8400 for RBFNN, for 5, 10 and 15 features, respectively. The linear SVM 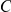 value was fixed to 1 by default. The SVM with polynomial and RBF kernels' 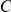 and 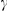 parameters produced the best classification performances. All 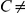 and 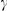 were determined into a heuristic way.

**Table 3:** MLP, RBF neural networks and linear SVM (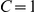), SVM with polynomial kernel (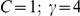) and SVM with RBF kernel (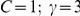) classifications using the top 5, top 10 and top 15 features selected via KS-test of the HRV records from normal (N) and cardiovascular risk (R) subjects.

#Features	Classifier		Se (%)	Sp (%)	Np (%)	Pp (%)	Ac (%)
5	MLP*	Training set	100.00	100.00	100.00	100.00	100.00
		Test set	93.33	100.00	93.75	100.00	96.67
	RBFNN*	Training set	96.67	100.00	96.67	100.00	98.33
		Test set	85.71	93.33	87.50	92.31	89.66
	SVM (Linear)	Training set	71.88	75.00	70.00	76.67	73.33
		Test set	72.73	72.73	72.73	72.73	72.73
	SVM (Polynomial kernel)	Training set	100.00	100.00	100.00	100.00	100.00
		Test set	84.09	70.45	81.58	74.00	80.00
	SVM (RBF kernel)	Training set	100.00	100.00	100.00	100.00	100.00
		Test set	74.24	78.79	75.36	77.78	76.52
10	MLP*	Training set	100.00	100.00	100.00	100.00	100.00
		Test set	86.67	80.00	85.71	81.25	83.33
	RBFNN	Training set	96.67	90.00	96.43	90.63	93.33
		Test set	60.00	100.00	71.43	100.00	80.00
	SVM (Linear)	Training set	100.00	100.00	100.00	100.00	100.00
		Test set	86.36	77.27	85.00	79.17	81.82
	SVM (Polynomial kernel)	Training set	100.00	100.00	100.00	100.00	100.00
		Test set	86.36	75.00	84.62	77.55	80.68
	SVM (RBF kernel)*	Training set	100.00	100.00	100.00	100.00	100.00
		Test set	96.97	81.82	96.43	84.21	89.39
15	MLP*	Training set	100.00	100.00	100.00	100.00	100.00
		Test set	73.33	100.00	78.95	100.00	86.67
	RBFNN	Training set	83.33	83.33	83.33	83.33	83.33
		Test set	60.00	100.00	72.43	100.00	80.00
	SVM (Linear)	Training set	100.00	100.00	100.00	100.00	100.00
		Test set	77.27	77.27	77.27	77.27	77.27
	SVM (Polynomial kernel)	Training set	100.00	100.00	100.00	100.00	100.00
		Test set	90.91	70.45	88.57	75.47	80.68
	SVM (RBF kernel)*	Training set	100.00	100.00	100.00	100.00	100.00
		Test set	93.94	77.27	92.73	80.52	85.61

*Classifiers that presented the higher performances on each experiment.

According to Table 3, the higher performance was reached by MLP using the top-5 features selected by KS-test. It is important to note that the performance of the classifiers was similar in all cases; therefore, many combinations of features are suitable for the prognosis of cardiovascular risk as it is proposed in our work. For the 5 optimal features, linear SVM selected 36 support vectors; polynomial kernel SVM selected 28 support vectors; and RBF kernel SVM selected 43 support vectors from the same training dataset.

In addition, as part of the experiments, we evaluated the effect of using all the features from all the analysis methods shown in this paper. The results showed that for SVM classifiers, the three schemes evaluated (linear, polynomial kernel and RBF kernel SVM) reached higher performances than ANN ones. The main results of these experiments are registered in Table 4. The results of ANN classification were not included due to the poorness of the classification performances, this is a common effect given by the overfitting produced by the high dimensionality of the input space.

**Table 4:** Results of linear SVM (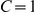), SVM with polynomial kernel (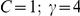) and SVM with RBF kernel (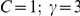) classifications using the total of features of the HRV records from normal (N) and cardiovascular risk (R) subjects.

Kernel		Se (%)	Sp (%)	Np (%)	Pp (%)	Ac (%)
Linear	Training set	100.00	100.00	100.00	100.00	100.00
	Test set	86.36	90.91	86.96	90.48	88.64
Polynomial	Training set	100.00	100.00	100.00	100.00	100.00
	Test set	79.55	86.36	85.37	80.85	82.95
RBF	Training set	100.00	100.00	100.00	100.00	100.00
	Test set	77.27	87.88	86.44	79.45	82.58

#### 2.2. Do Multi-resolution and Non-linear Features Perform Better than Conventional Statistical and Spectral Features?

A topic of remarkable discussion has been whether non-linear and multi-resolution features on HRV analysis perform better than the conventional and clinical-applied statistical and spectral analysis methods [2], [27], [28], [29], [30], [31]. On the basis of determining the real usefulness of the non-linear and multi-resolution features in terms of the prognostic value (Se, Sp, Pp, Np and Ac), extensive experiments were carried out in this work.

The proposed classifiers were trained using statistical and spectral features (classical analysis features) and using the multi-resolution and non-linear features (novel features) separately, especially in order to know the overall suitability of each group of features with respect to the HRV analysis. The main results of our experiments are depicted in Table 5. The ANN and SVM schemes were implemented under the same setup as described in the previous section. When statistical and spectral features were used to classify the data, the number of support vectors chosen by linear SVM, polynomial kernel SVM and RBF kernel SVM was 36, 24 and 46, respectively. On the other hand, when multi-resolution and non-linear features were used, linear SVM, polynomial kernel SVM and RBF kernel SVM selected 30, 24 and 46 support vectors, respectively.

**Table 5:** MLP, RBF neural networks and linear SVM (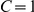), SVM with polynomial kernel (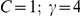) and SVM with RBF kernel (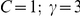) classifications using classical and non-linear/multi-resolution features of the HRV records from normal (N) and cardiovascular risk (R) subjects.

Features	Classifier	Se (%)	Sp (%)	Np (%)	Pp (%)	Ac (%)
Statistical + Spectral	MLP	66.67	60.00	64.29	62.50	63.33
	RBFNN	26.67	93.33	56.00	80.00	60.00
	SVM (Linear)	72.73	86.36	76.00	84.21	79.55
	SVM (Polynomial kernel)	68.18	70.45	68.89	69.77	69.32
	SVM (RBF kernel)	68.18	74.24	70.00	72.58	71.21
Non-linear + Multi-resolution	MLP	80.00	100.00	83.33	100.00	90.00
	RBFNN	73.33	100.00	78.95	100.00	86.67
	SVM (Linear)	95.45	77.27	94.44	80.77	86.36
	SVM (Polynomial kernel)	88.64	81.82	87.80	82.98	85.23
	SVM (RBF kernel)	90.91	83.33	90.16	84.51	87.12

Table 5 illustrates at least two important findings from the experiments carried out for this part of the research: First, non-linear and multi-resolution features have remarkably higher prognostic value than the features referred to as classical analysis (this fact was also confirmed by the statistical significance of the features already reported in this work); and second, the results indicate indirectly the overall usefulness of certain combinations of statistical and spectral measures and their expected effectiveness in clinical applications (it is important to note that even when these classical features are not very suitable to HRV analysis, the non-linear nature of the classifiers is the main catalyst to reach moderately good performances).

Due to the non-linearity and non-stationarity of the HRV signal, many authors prefer using non-linear and multi-resolution features rather than statistical or spectral ones in order to train classifiers [20], [21]. Nonetheless, it is important to note that as the complexity of the features increases, also its medical interpretation becomes obscure and cumbersome. Furthermore, especially multi-resolution decomposition produces an important loss on the potential interpretation of features and signals, regardless of the remarkable increase of the statistical significance of the features reported using such analyses.

#### 2.3. PCA-based Feature Selection Results

In addition to the results reported above, several classification experiments regarding PCA features' projections were performed in our work. They were carried out using the cross validation method under the same setup reported above. These experiments were made on the basis of two main goals: First, in order to investigate whether the significance levels of the projected features remained similar to the original ones; and second, to classify the data using the 3, 4, 5 and 10 principal features' projections from such PCA analysis, as the variance retained by the 3, 4, 5 and 10 first components for both groups –normal healthy subjects and cardiovascular risk patients– was equal to 77.65%, 83.33%, 86.96% and 94.45%, respectively. Furthermore, the 99% of the variance of the model was retained by the first 21 components. According to the KS-test results, from the five first PCA projections, three of them remained to be statistically significant (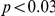), and in one case, the seventh PCA projection also showed high statistical significance (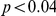); the rest of the components were not statistically significant for the comparison of normal (N) and cardiovascular risk (R) subjects.

The results of the ANN and SVM schemes classification of HRV records from normal (N) and cardiovascular risk (R) subjects are depicted in Table 6. With respect to the classification results using the projected features, linear SVM selected 46, 32, 27 and 23 support vectors from the whole training dataset; polynomial kernel SVM chose 28, 22, 21 and 21 support vectors; and RBF kernel SVM picked up 46 support vectors in all cases. Table 6 clearly illustrates important improvements for the performance of SVM-based classifiers. On the other hand, MLP and RBFNN schemes presented highly limited performances over the entire track of experiments regarding the implementation of PCA. However, the greatest performance reported by our work was achieved by MLP when the KS test-based feature selection was performed.

**Table 6:** MLP, RBF neural networks and linear SVM (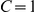), SVM with polynomial kernel (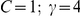) and SVM with RBF kernel (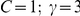) classifications using the projections of the features from PCA analysis of the HRV records from normal (N) and cardiovascular risk (R) subjects.

Number of PCA Projections	Classifier	Se (%)	Sp (%)	Np (%)	Pp (%)	Ac (%)
3	MLP	60.00	73.33	64.71	69.23	66.67
	RBFNN	40.00	93.33	60.87	85.71	66.67
	SVM (Linear)	77.27	63.64	73.68	68.00	70.45
	SVM (Polynomial kernel)	84.09	70.45	81.58	74.00	77.27
	SVM (RBF kernel)	89.39	65.15	86.00	71.95	77.27
4	MLP	80.00	80.00	80.00	80.00	80.00
	RBFNN	46.67	86.67	61.90	77.78	66.67
	SVM (Linear)	90.91	90.91	90.91	90.91	90.91
	SVM (Polynomial kernel)	90.91	93.18	91.11	93.02	92.05
	SVM (RBF kernel)	93.94	89.39	93.65	89.86	91.67
5	MLP	66.67	100.00	75.00	100.00	83.33
	RBFNN*	N/A	N/A	N/A	N/A	N/A
	SVM (Linear)	86.36	95.45	87.50	95.00	90.91
	SVM (Polynomial kernel)	81.82	95.45	84.00	94.74	88.64
	SVM (RBF kernel)	81.82	95.45	84.00	94.74	88.64
10	MLP	80.00	73.33	78.57	75.00	76.67
	RBFNN*	N/A	N/A	N/A	N/A	N/A
	SVM (Linear)	81.82	81.82	81.82	81.82	81.82
	SVM (Polynomial kernel)	81.82	95.45	84.00	94.74	88.64
	SVM (RBF kernel)	81.82	95.45	84.00	94.74	88.64

*The results for this classifier were not reported due to their poorness.

The main aim of using these four different numbers of PCA projections was to compare the performance reached by the classifiers and to identify whether the data structure was suitable for the proposed classifiers architecture. Indeed, there exists more than one group of features that reported high success rates at classifying the HRV records. Amongst all the classifiers, the maximum overall performance was reached by the polynomial kernel SVM with overall sensitivity, specificity and accuracy of 90.91%, 93.18% and 92.05%, respectively.

## Discussion

Heart rate variability has been related to numerous cardiac diseases. According to the literature, the short variations on statistical features of the NN intervals are related to: Complete heart block (CHB), left bundle branch block (LBBB) and ischemic cardiopaty. On the other hand, the long variations on statistical features of the NN intervals are related to: Premature ventricular contractions (PVCs), sinus syndrome (SSS) and atrial fibrillation (AF). There are several modifications on AR spectrums that can be noticed by the calculation of the power in frequency bands. Short variations of HRV segments usually lead to high VLF and LF bands power. Conversely, long variations of HRV segments, usually leads to higher HF band power. As cardiovascular risk is highly related to variations on statistical and spectral components, one of the major disadvantages of these methods is the linearity and consequently, their poor suitability for highly non-stationary HRV dynamics and, certainly, the NN intervals fluctuation by nervous mechanisms. The Fourier transform techniques (frequency domain methods such as Welch periodogram or AR spectrum as well) resolve the time domain signal into complex exponential functions, along with information about their phase shift measured with respect to a specific reference instant. Here the frequency components extend from 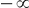 to 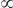 in the time scale. That is, even finite length signals are expressed as the sum of frequency components of infinite duration. Besides, the phase angle, being a modular measure, fails to provide the exact location of an ‘event’ along the time scale. This is a major limitation of the Fourier transform approach [2]. Thereby, considering the cardiovascular system as nonlinear in nature, can lead to a better understanding of its dynamics.

The patterns in HRV are directly related to the Poincaré map patterns in visual assessment. In the case of short fluctuations, HRV segments are not much dispersed and torpedo shapes [62] are predominant in many cases. In the case of long fluctuations, HRV segments appear to be very dispersed forming complex-like and fan-like return map shapes. All these Poincaré plot patterns are directly related with cardiovascular risk [66].

One of the main contributions of this work is the prognosis of cardiovascular risk in a general way, not only for specific cardiac diseases or the prediction of specific cardiac episodes like other publications have shown [1], [8], [13], [14], [16], [18], [19], [20], [21], [29], [55], [61]. Moreover, it has been confirmed that multi-resolution and non-linear analysis are much more suitable for the assessment and prognosis of cardiovascular risk than statistical and spectral classical analysis. KS significance tests also confirmed that those features lead to higher statistical significance levels (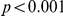 for the case of ApEn and SmEn features). We developed a method that combines statistical, spectral, multi-resolution and non-linear features as well as ANN and SVM schemes for the prognosis of cardiovascular risk. Exactly 90 HRV records were analyzed, 60 of them were used to train and 30 to test each classification scheme.

From the classification schemes, MLP provided the best classification rates for the prognosis of cardiovascular risk, with an area under the ROC curve equal to 0.9800. On the other hand, schemes such as the RBF network and SVMs showed relatively high classification performances too. Another remarkable finding is the improvement of the classification rates of SVM using all the extracted features (not only the features selected by KS significance tests) and PCA, which can be attributable to the computation of the decision surface and the apparent SVM bias towards the positive and negative cases. Furthermore, as expected, ANN schemes often presented overfitting using all those features. The comparison between the performances of all the implemented classifiers was limited to sensitivity, specificity, positive predictive value, negative predictive value and accuracy as a consequence of ROC curves limitations, i.e., usually its contributions become cumbersome when a comparison between different classifiers is needed. Besides, their transformation to objective values is usually limited to the calculation of the area under the curve [67].

According to findings reported in the literature [5], breath rate modifies the fluctuation of the NN intervals in a HRV sample record, i.e., there is an evident modification of the HRV when the analyzed subject is breathing at different frequencies (e.g., 6 breaths/min or 12 breaths/min) provided that the rhythmic fluctuations can be larger or shorter given the regulation mechanisms and the RSA dynamics as an effect of the activity of neural oscillators. As a direct consequence, the experimental results from the comparison between records at different breathing rates would be invalid. Thus, another contribution of this work is the consideration of the breathing rate as an additional variable for the assessment of HRV; this feature was included in every record taken for the HRV database reported in this work.

Besides the strong considerations of the breathing rate and the breathing signal, our computational implementation allows working with the ECG and HRV signals for medical analysis. There are new possibilities of analysis that commercial and conventional HRV analysis software [55] have not yet considered; additionally, there is the possibility of performing an automatic prognosis using a trained ANN scheme embedded in the program. The main objective of this module is to give support to the specialist criteria on the HRV assessment. This computational application needs strong validation and medical feedback, which will be a topic for future research.

According to the results of this study, we strongly suggest working with multi-resolution and non-linear analysis in order to achieve more reliable cardiovascular risk prognosis, especially for classification schemes such as ANNs and SVMs. It is evident that a nonlinear deterministic approach is more appropriate to describe more complex phenomena, indicating that apparently erratic behavior can be generated even by a simple deterministic system with nonlinear structure. In general terms, the fluctuations of heartbeats during normal sinus rhythm could be partially attributed to deterministic chaos, and a decrease in this type of nonlinear variability could be observed in different cardiovascular diseases [58]. Further investigation is needed for the incorporation of chaotic dynamics and fractals to the analysis of HRV for the prognosis of cardiovascular risk proposed in this study.

### Conclusions

This work has been focused on 4 major aspects: (1) The HRV database conformation integrating the breathing signal; (2) integrating the breathing frequency to the analysis of HRV records; (3) the analysis of statistical, spectral, multi-resolution and non-linear features linked with classification schemes for the prognosis of cardiovascular risk as well as the confirmation of the properties of these features as they are reported in the literature; (4) a brief illustration of the software implementation of advanced HRV analysis integrating an automatic prognosis tool using a standard 5 min ECG record.

This work has developed a method for the cardiovascular risk prognosis using statistical, spectral, multi-resolution and non-linear features extracted from HRV data. The database used in this work was recorded using a proper medical protocol and contains a total of 90 HRV and breathing signal records from normal and cardiovascular risk subjects. The suitability of each HRV analysis feature has been shown by using KS-tests and neural and support vector classifiers. Short-term HRV features are highly useful for the prognosis of cardiovascular risk; nonetheless, long-term features can be useful for increasing the positive predictive value of the proposed classifiers.

In addition, it has become obvious that the performance reported by the different feature selection strategies depends largely on the pattern recognition scheme implemented to classify the data. For the short-term HRV features analyzed in this work, there were two main observed effects: First, the ANN schemes were more suitable for the KS test-based feature selection as well as for feature spaces of low dimension; and second, SVM schemes were more suitable for the PCA-based feature selection as well as to high dimensional feature spaces. Nonetheless, as the PCA projected features improved by far the performance of SVM classifiers, their statistical significance showed influential decrease that affected negatively the performance of the ANN classifiers.

Breathing signal and breathing frequency were employed in this work for the analysis of HRV, given the findings about RSA phenomena on the modification of the fluctuations of NN intervals at different breathing frequencies [5]. All the HRV measurements in this work were done approximately at 12 breaths/min for every single subject in the database; this was guaranteed by the procedures established in the medical protocol that we designed to carry out this research; moreover, the acquired breathing signals confirmed it.

Finally, HRV signal –as both a traditional and non-linear signal in nature– has been an important predictor of cardiovascular risk. Furthermore, this work represents an important step in comprehensively understanding such dynamics, and more importantly, on the prognosis of cardiovascular risk as a stated goal established by Chattipakorn et al. (2007) and a plethora of researchers and clinicians: “It is necessary to understand the mechanisms of HRV components and improve their sensitivity and specificity as a prognostic marker” [68]. Furthermore, the combination of the methods reported in this work and other ECG and physiological parameters is expected to lead to a solution for the prognosis of cardiovascular mortality at reasonable cost-effectiveness.

## Supporting Information

Appendix S1 **Detailed description of complexity measures calculation.**
(DOC)

Appendix S2 **Detailed description of SVM learning algorithm.**
(DOC)
