# Supplementary material for: Heart Rate Variability Dynamics for the Prognosis of Cardiovascular Risk
Source: PLoS One. 2011 Feb 28;6(2):e17060. doi: 10.1371/journal.pone.0017060 (PMC3046173; doi:10.1371/journal.pone.0017060)
Supplement: Appendix S1 — Detailed description of complexity measures calculation. (DOC) [file pone.0017060.s001.doc]

# Appendix S1

Let $\left\{x\left(n\right)\right\}$ represents a time-series of length *N*. Consider the *m*-length vectors: $X(1)...X(N-m+1)$, with $X(i)=\left[x(i),x(i+1),...,x(i+m-1)\right]$, $1\leq i\leq N-m+1$. These vectors represent *m* consecutive values of *x*. Let us define the distance between $X(i)$ and $X(j)$:

$d\left[X(i),X(j)\right]=\max_{k=1,...,m} \left(\left|x\left(i+k\right)-x\left(j+k\right)\right|\right)$. (1)

Let $N^{m}(i)$represents the number of $j$ $\left(1\leq j\leq N-m+1,j\neq i\right)$ that satisfies $d\left[X(i),X(j)\right]\leq r$. Then, the probability that any vector $X(i)$is close to the vector $X(j)$ is given by:

$C_{r}^{m}(i)=\frac{N^{m}(i)}{(N-m+1)}$. (2)

The average of this equation represents the probability that any two vectors of length *m* are within the radius *r*:

. (3)

$C_{r}^{m+1}(i)$ and $\varphi^{m+1}(r)$should be computed following the same nomenclature and the same procedure. Then ApEn can be obtained using the following equation:

$ApEn\left(m,r\right)=\varphi^{m}(r)-\varphi^{m+1}(r)$. (4)

On the other hand, SmEn has the advantage of being less dependent on time-series length than ApEn, and shows a relative consistency over a broader range of possible *r*, *m* and *N* values [1]. Moreover, SmEn is defined by:

$SmEn(m,r)=-\ln\left[\frac{U^{m}(r)}{U^{m+1}(r)}\right]$. (5)

## References

1. Chesnokov YV (2008) Complexity and spectral análisis of the heart rate variability dynamics for distant prediction of paroxysmal atrial fibrillation with artificial intelligence methods. Artificial Intelligence in Medicine 43: 151-165
