# Supplementary material for: Heart Rate Variability Dynamics for the Prognosis of Cardiovascular Risk
Source: PLoS One. 2011 Feb 28;6(2):e17060. doi: 10.1371/journal.pone.0017060 (PMC3046173; doi:10.1371/journal.pone.0017060)
Supplement: Appendix S2 — Detailed description of SVM learning algorithm. (DOC) [file pone.0017060.s002.doc]

# Appendix S2

Given a training data set $\left(x_{i},d_{i}\right)$, it is not possible to find a separation hyperplane without trading with classification errors [1]. As a consequence of this problem a new set of non-negative scalar variables is introduced $\left\{\xi\right\}_{i=1}^{N}$, on the definition of separation hyperplane.

Thereby, the way to find the separation hyperplane in order to minimize the classification error, is minimizing the following simplified equation:

$\varphi\left(w,\xi\right)=\frac{1}{2}w^{T}w+C\sum_{i=1}^{N} \xi_{i}$, (1)

subjected to: $d_{i}\left(w^{T}x_{i}+b\right)\geq1-\xi_{i}$, for $i=1,2,...,N$,

where *w* is the weight vector, subjected to the restriction given in (20), the $\xi_{i}$ measures the deviation of a data point from the ideal condition of pattern separability, *b* is the bias variable and *C* is a user-defined parameter.

By using the Lagrange multipliers method, it is possible to formulate the actual problem as the following equation expresses:

$Q\left(\alpha\right)=\sum_{i=!}^{N} \alpha_{i}-\frac{1}{2}\sum_{i=1}^{N} \sum_{j=1}^{N} \alpha_{i}\alpha_{j}d_{i}d_{j}x_{i}^{T}x_{j}$. (2)

This equation is the simplified version of the Lagrangian minimized with respect to the primal variables *w* and *b* and maximized with respect to the dual variables $\alpha$.

subjected to the following conditions:

$\sum_{i=1}^{N} \alpha_{i}d_{i}=0$, (3)

and:

$0\leq\alpha_{i}\leq C$, for $i=1,2,...,N$ (4)

On the other hand, the optimal solution of the weight vector is given by the following equation:

$w_{0}=\sum_{i=1}^{N_{s}} \alpha_{0,i}d_{i}x_{i}$, (5)

where *Ns* is the number of support vectors.

The way to determine the optimal values of the biases is given by the following expressions:

$\alpha_{i}\left[d\left(w^{T}x_{i}+b\right)-1+\xi_{i}\right]=0$, for $i=1,2,...,N$ (6)

and

$\mu_{i}\xi_{i}\approx0$, for $i=1,2,...,N$ (7)

where $\mu_{i}$are the Lagrange multipliers which have been introduced to enforce the non-negativity of $\xi_{i}$.

### Kernelization

The variation between a SVM and a K-SVM, is the need to express the mathematical equations in terms of dot products between the features and these features in terms of the kernel *k*, evaluated for the input patterns as the following equation describes:

$k\left(x,x'\right)=\left(x\cdot x'\right)$. (8)

Thereby, decision functions can be obtained in a general form [2]:

$f\left(x\right)=sign\left(\sum_{i=1}^{m} \alpha_{i}d_{i}\cdot k\left(x,x_{i}\right)+b\right)$. (9)

And therefore, the term to maximize, with the constant *C* set to be equal to one, is illustrated by the following equations:

$W\left(\alpha\right)=\sum_{i=1}^{m} \alpha_{i}-\frac{1}{2}\sum_{i,j=1}^{m} \alpha_{i}\alpha_{j}d_{i}d_{j}k\times\left(x_{i},x_{j}\right)$, (10)

subjected to

$\alpha_{i}\geq0$; $i=1,...,m$ and $\sum_{i} \alpha_{i}d_{i}=0$. (11)

This equation is the simplified version of the Lagrangian minimized with respect to the primal variables *w* and the bias *b* and maximized with respect to the dual variables $\alpha$.

## References

1. Haykin S (1999) Neural Networks: A Comprehensive Foundation. Prentice Hall, inc. p. 823.
2. Wang L, Liu B, Wan C (2005) Classification using support vector machines with graded resolution. Proceedings of the international conference on granular computing: 666-670
